# Management of epilepsy in resource-limited settings: establishing an epileptic surgery program in Southern Iran

**Published:** 2012-11

**Authors:** Ali Akbar Asadi-Pooya, Mousa Taghipour, Ahmad Kamgarpour, Seyed Mohammad Rakei, Ali Razmkon

**Affiliations:** ^a^Shiraz Neurosciences Research Center, Shiraz University of Medical Sciences, Shiraz, Iran.

## Abstract

**Background:**

The most common treatment modality for epileptic patients is antiepileptic drug (AED) therapy. However, more than 30% of epileptic patient suffers from drug-resistant epilepsy despite the use of appropriate AEDs. Surgery is a common option for these patients. Epilepsy surgery requires sophisticated medical technologies, many of them not available in Iran. The authors of the present study aim to report the clinical and technical issues of establishing an epilepsy surgery protocol in Iran and the limitations and recommendations for further development.

**Methods:**

Medically-refractory epileptic patients underwent common imaging techniques (mainly brain MRI using a specific temporal lobe protocol) as well as continuous video EEG monitoring. A specialized committee consisting of epileptologists, neurologists, neurosurgeons and radiologists discussed the eligibility of each patient, and defining treatment plans for the surgery.

**Results:**

A total of 22 committee sessions were hold to discuss 140 cases with medically-refractory seizures during March 2009 to May 2012. Eighty-eight patients (62%) underwent the surgery, 35 patients (25%) were considered not appropriate for surgery, and 17 patients (12%) refused to undergo surgery. The most common cause of intractable seizures was mesial temporal sclerosis (36%), brain tumors (21%), cortical malformations (6%), and encephalomalacia (4%). Among 81 operated patients, 39.5% had anterior temporal lobectomy, 34.5% corpus callosotomy, and 30% underwent lesionectomy.

**Conclusions:**

The success of epilepsy surgery depends on the accurate identification of good surgical candidates based on available resources and technologies without jeopardizing safety. The key step in establishing a successful plan is having access to trained epileptologist and neurosurgeon.

**Keywords:**

Epileptology, Epilepsy surgery, Antiepileptic drug, Surgery portocol


					Volume 4, Suppl. 1 Nov 2012
Publisher: Kermanshah University of Medical Sciences
URL: http://www.jivresearch.org
Copyright:
Congress of Iranian Neurosurgeons, 14-16 November 2012, Kermanshah, Iran
Paper No. 16
Management of epilepsy in resource-limited settings:
establishing an epileptic surgery program in Southern Iran
Ali Akbar Asadi-Pooya a
, Mousa Taghipour a
, Ahmad Kamgarpour a
, Seyed-Mohammad Rakei a
,
Ali Razmkon a,*
a
Shiraz Neurosciences Research Center, Shiraz University of Medical Sciences, Shiraz, Iran.
Abstract:
Background: The most common treatment modality for epileptic patients is antiepileptic drug (AED) therapy.
However, more than 30% of epileptic patient suffers from drug-resistant epilepsy despite the use of appropriate
AEDs. Surgery is a common option for these patients. Epilepsy surgery requires sophisticated medical technologies,
many of them not available in Iran. The authors of the present study aim to report the clinical and technical issues of
establishing an epilepsy surgery protocol in Iran and the limitations and recommendations for further development.
Methods: Medically-refractory epileptic patients underwent common imaging techniques (mainly brain MRI using a
specific temporal lobe protocol) as well as continuous video EEG monitoring. A specialized committee consisting of
epileptologists, neurologists, neurosurgeons and radiologists discussed the eligibility of each patient, and defining
treatment plans for the surgery.
Results: A total of 22 committee sessions were hold to discuss 140 cases with medically-refractory seizures during
March 2009 to May 2012. Eighty-eight patients (62%) underwent the surgery, 35 patients (25%) were considered
not appropriate for surgery, and 17 patients (12%) refused to undergo surgery. The most common cause of
intractable seizures was mesial temporal sclerosis (36%), brain tumors (21%), cortical malformations (6%), and
encephalomalacia (4%). Among 81 operated patients, 39.5% had anterior temporal lobectomy, 34.5% corpus
callosotomy, and 30% underwent lesionectomy.
Conclusions: The success of epilepsy surgery depends on the accurate identification of good surgical candidates
based on available resources and technologies without jeopardizing safety. The key step in establishing a successful
plan is having access to trained epileptologist and neurosurgeon.
Keywords:
Epileptology, Epilepsy surgery, Antiepileptic drug, Surgery portocol
* Corresponding Author at:
Ali Razmkon: Shiraz Neuroscience Research Center, Shiraz University of Medical Sciences, Shiraz, Iran, Tel: 09173148711
Email: Ali.razmkon@gmail.com, (Razmkon A.).

				

